# Development and validation of a novel deep learning coronary artery plaque quantification model

**DOI:** 10.1186/s12880-026-02379-z

**Published:** 2026-05-14

**Authors:** Johan Malmqvist, Tomas Jernberg, Ramtin Vedad, Chunliang Wang

**Affiliations:** 1 Department of Clinical Sciences, Danderyd Hospital, Karolinska Institutet, Stockholm, 182 88 Sweden; 2 Department of Biomedical Engineering and Health Systems, KTH Royal Institute of Technology, Stockholm, Sweden

**Keywords:** Atherosclerosis, Deep-learning, CCTA, Validation, Plaque volume

## Abstract

**Background:**

Coronary Computed Tomography Angiography (CCTA) is an established tool for assessing coronary artery disease. CCTA determined total coronary artery plaque volume predicts cardiovascular events, but manual quantification is impractical for routine use. Deep learning-based methods offer a promising solution for automated plaque volume assessment.

**Methods:**

We developed and validated a novel software, *QuantiPlaque*, powered by deep learning models, for automated segmentation of total and calcified coronary plaque volume. Expert manual annotation served as the reference standard. Correlations between deep learning and expert segmentation were evaluated using intraclass correlation coefficient (ICC), Pearson’s r, and Spearman’s rho, and agreements were evaluated with Bland-Altman analyses.

**Results:**

A total of 115 CCTA scans were included. Mean (range) age was 58 (51–64) and 51% were females. The mean coronary artery calcium score was 56 Agatston units, ranging from 0 to 601. The model demonstrated strong correlation and agreement with expert annotation for per-patient total plaque volume (ICC 0.95, mean difference − 8.35 mm^3^, 95% limit of agreement − 102 to 85 mm^3^, Spearman’s rho 0.87, Pearson’s r 0.95) and calcified plaque volume (ICC 0.90, mean difference − 0.28 mm^3^, 95% limits of agreement − 21.97 to 21.42 mm^3^, Spearman’s rho 0.93, Pearson’s r 0.90). Per-vessel analysis showed strong correlation in the left anterior descending artery and right coronary artery but was weaker in the left circumflex artery territory.

**Conclusion:**

The evaluated deep learning model provides accurate quantification of total and calcified plaque burden, with strong correlation and agreement to expert annotation.

## Introduction

Coronary Computed Tomography Angiography (CCTA) has become a widely available non-invasive method for diagnosing flow-limiting coronary artery stenoses as well as non-obstructive coronary atherosclerosis. Over the past decades, CCTA has rapidly advanced both technically and in its growing body of evidence for cardiovascular risk assessment [1]. Both obstructive and non-obstructive stenoses (obstruction typically defined as > 50% lumen narrowing) have been shown to predict total mortality as well as major cardiovascular events [2, 3]. Quantitative metrics of coronary atherosclerotic disease such as total plaque volume have also been shown to predict cardiovascular outcomes [4–6], but manually quantifying total plaque volume is too time consuming to be done routinely and indirect measures of plaque burden such as segment involvement score (SIS), coronary artery calcium score (CACS) or visual assessment are often used in clinical practice and research [7]. Deep learning algorithms have the potential to rapidly and accurately quantify both calcified and non-calcified plaque burden in the coronary arteries, improving cardiovascular risk prediction and personalized treatment planning. However, it is essential that such new tools are carefully validated before implementation. We aimed to develop and externally validate a new deep learning model for quantification of coronary artery total plaque volume and calcified plaque volume.

## Methods

**Fig. 1:**
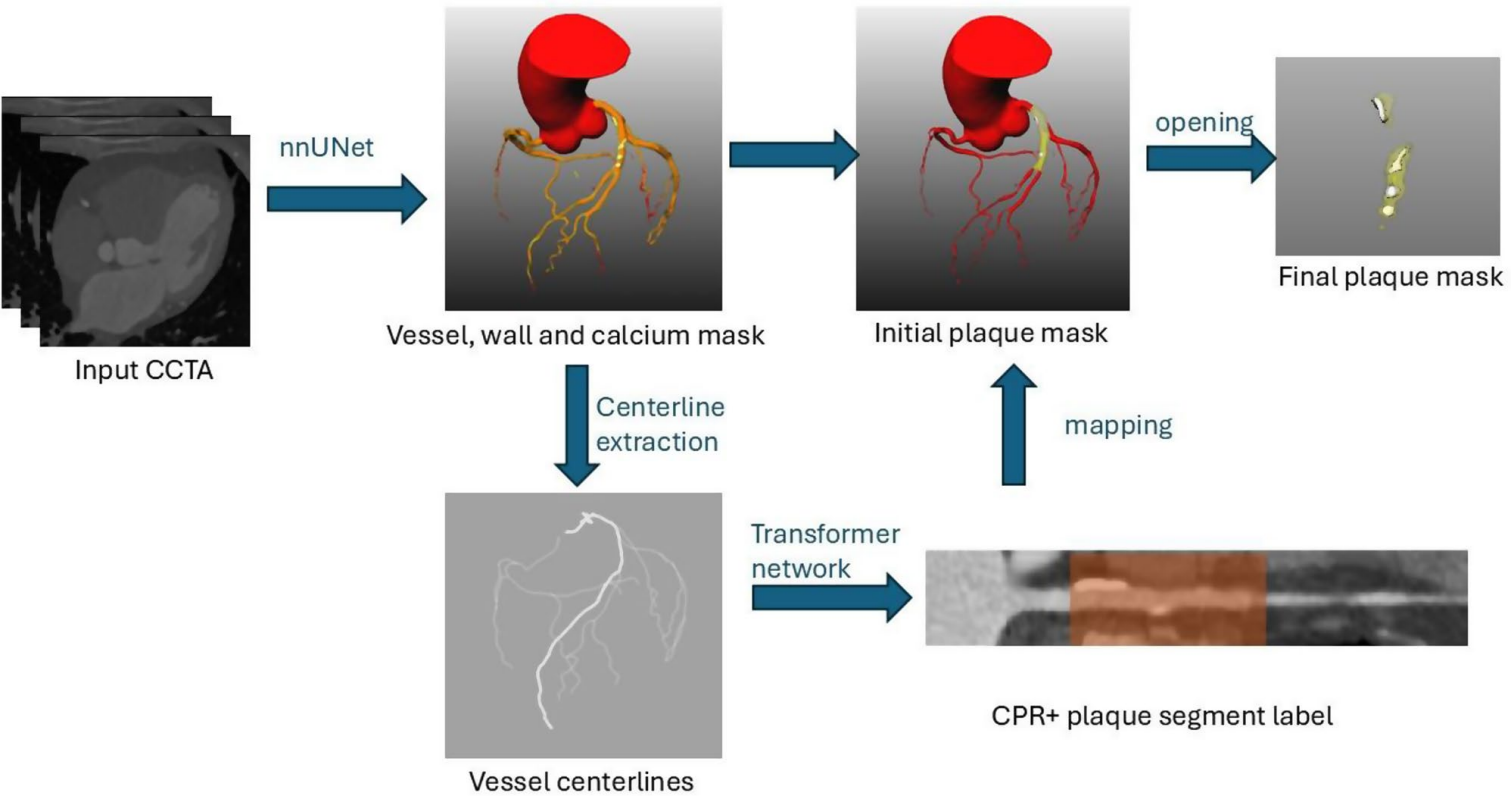
Overview of the image processing pipeline in QuantiPlaque

A novel software, *QuantiPlaque*, powered by deep learning networks trained on a CCTA dataset with images from various CT scanners and scanning protocols, was developed. The overall processing pipeline is summarized in Fig. 1. Plaque segmentation is performed using two 3D deep learning networks. The first network segments the coronary artery tree, including the vessel wall and calcified regions, from CCTA volumes. This is followed by a second network that identifies vessel segments with visible plaques along the centerlines. The vessel wall is then constrained to the detected regions, and the final plaque mask is generated using a morphological opening operation to remove the healthy vessel wall, which is typically much thinner.

Built on the nnUNet framework, the first segmentation network was trained on 860 CCTA scans. This dataset included 500 publicly available cases from the ImageCAS database acquired on a Siemens 128-slice dual-source scanner, as well as an in-house collection comprising 160 scans from a GE Discovery system, 140 from a Definition Flash scanner, and 60 from a Philips iCT. All images were normalized using the default CT preprocessing pipeline in nnU-Net. By design, nnU-Net trains five U-Net models using 5-fold cross-validation (80:20 training–validation split per fold), and the model achieving the best validation performance was selected as the final model. To maximize the use of available data, no independent test set was reserved for final training. In contrast to the standard nnUNet configuration, a Connectivity-Preserving Dice Loss [8] was employed to reduce the risk of segmenting the vessel tree into disconnected parts. This loss function strongly penalizes topological errors during vessel segmentation, such as fragmentation of a vessel into multiple segments, thereby promoting anatomical continuity. Further technical details and evaluation can be found in our previous work [8]. The nnUNet network produces labels for coronary artery, vessel wall and calcified coronary plaques. Ground truth vessel and plaque segmentations were first generated using an in-house interactive coronary segmentation tool [9, 10] and subsequently refined in ITK-SNAP. This tool combines a skeleton-based shape model with a level-set method, enabling rapid extraction of vessel centerlines and semi-automatic delineation of inner and outer vessel wall surfaces via global and local threshold adjustments, rather than manual slice-by-slice annotation. Further manual corrections and calcified plaque labeling were performed in ITK-SNAP by trained medical engineers and verified by a senior radiologist with over ten years of CCTA experience.

Following segmentation, centerlines of the extracted coronary arteries are computed via skeletonization. Based on the extracted centerlines, straightened curved planar reformation (CPR) images are generated for each centerline. A 3D vision transformer network, adapted from an open-source implementation [11] and trained on 671 coronary branches from 402 patients, then analyzes the straightened volumes, detecting regions with visible plaque and determining their start and end points. The transformer processes the straightened vessel as an array of 3D patches and predicts, at the slice level, whether plaque is present, without requiring voxel-wise plaque segmentation. The network outputs a one-dimensional array indicating plaque presence per slice. Training was performed with a 9:1 training–validation split, using ground truth plaque annotations generated in ITK-SNAP on the straightened volumes.

Finally, the stenotic segments are mapped back onto the original CT volume to identify vessel wall voxels within these regions. The resulting intersection mask is then upsampled to an isotropic resolution of 0.20 mm, followed by a morphological opening operation with a spherical kernel of 0.4 mm to remove residual healthy vessel wall. Plaque volumes are subsequently segmented according to coronary artery territories: right coronary artery (RCA), left main/ left anterior descending artery (LM/LAD) and left circumflex artery (LCX). The territories are determined by analysing the vessel centrelines extracted in the previous steps. The origins of the centrelines are used to separate right coronary arteries from the rest. While the first major bifurcation is used to determine the LM, LAD and LCX branches.

For both networks, an iterative annotation–training strategy was employed to reduce annotation workload. Initial manual annotations were used to train preliminary models, which were subsequently leveraged to generate additional labeled data for further refinement.

A dataset composed of 115 CCTA scans from the Swedish SCAPIS observational cohort was used for external validation of the deep learning software. No CCTA scans from the SCAPIS cohort had been used for training of the evaluated deep learning networks. Scans for the validation dataset were selected based on CACS to represent a wide range of atherosclerotic disease burden. All scans were performed using an ECG-triggered dual-source CT scanner equipped with a Stellar Detector (Somatom Definition Flash, Siemens Medical Solutions). Intravenous and/or oral metoprolol was administered to reduce heart rate below 60 beats/minute. Study subjects with systolic blood pressure more than 110 mmHg were given sublingual glyceryl nitrate. Contrast medium iohexol was administered (32 mg I/kg, injection time 12 s). Scanning protocols used included flash spiral, sequential and spiral. Tube voltages were either 100 kV or 120 kV according to body weight and heart rhythm [12]. 

The accuracy of the fully automatic deep learning workflow was validated against manual plaque delineation performed by a single level II CCTA-certified cardiologist (J.M.). CT scans were manually screened for major artifacts, and these cases were excluded from analysis. After automatic coronary artery centreline extraction, coarse delineation of inner and outer lining of artery wall was produced by Hounsfield Unit thresholds. Inner and outer wall delineations were subsequently manually adjusted one vessel branch at a time. The adjustments were made in long- and short axis curved planar reformation images of the coronary artery branches, and the reader was allowed to adjust image window level and width. The lengths of any visible plaques were outlined by manually marking the proximal and distal edges of each plaque. Parts of the marked segments’ circumference with wall thickness exceeding 0.8 mm were classified as plaque; any vessel wall thinner than that were excluded from the plaque mask volume by the same morphological opening operation as for the automatic pipeline. Calcified plaque volume was annotated pixel-by-pixel in axial view using the brush tool of the open-source software ITK-SNAP version 3.8.0. A level III CCTA certified cardiologist (R.V.) annotated 15 cases for analysis of interobserver variability.

Plaque volumes were treated as continuous variables and statistics used were intraclass correlation coefficient (ICC), Pearson’s r, Spearman´s rho, correlation plots and Bland-Altman plots to examine agreement. Fisher’s transformation was used for calculating 95% confidence intervals. R programming language was used for statistical analysis.

The study has been approved by the Swedish Ethical Review Authority (Dnr 2023-02237-01) and written informed consent has been obtained from all study participants. All methods were carried out in accordance with the Declaration of Helsinki.

## Results

The SCAPIS study participants used for external validation ranged in age from 51 to 64 years, with a mean age of 58 years. 51% were women. Mean CACS was 56 Agatston units ranging from 0 to 601. Sixteen cases (14%) were excluded from analysis due to major step artifacts. Ninety-nine CCTA scans were analysed. The mean time required for manual annotation was 33 min ranging from 12 to 70 min.

**Fig. 2:**
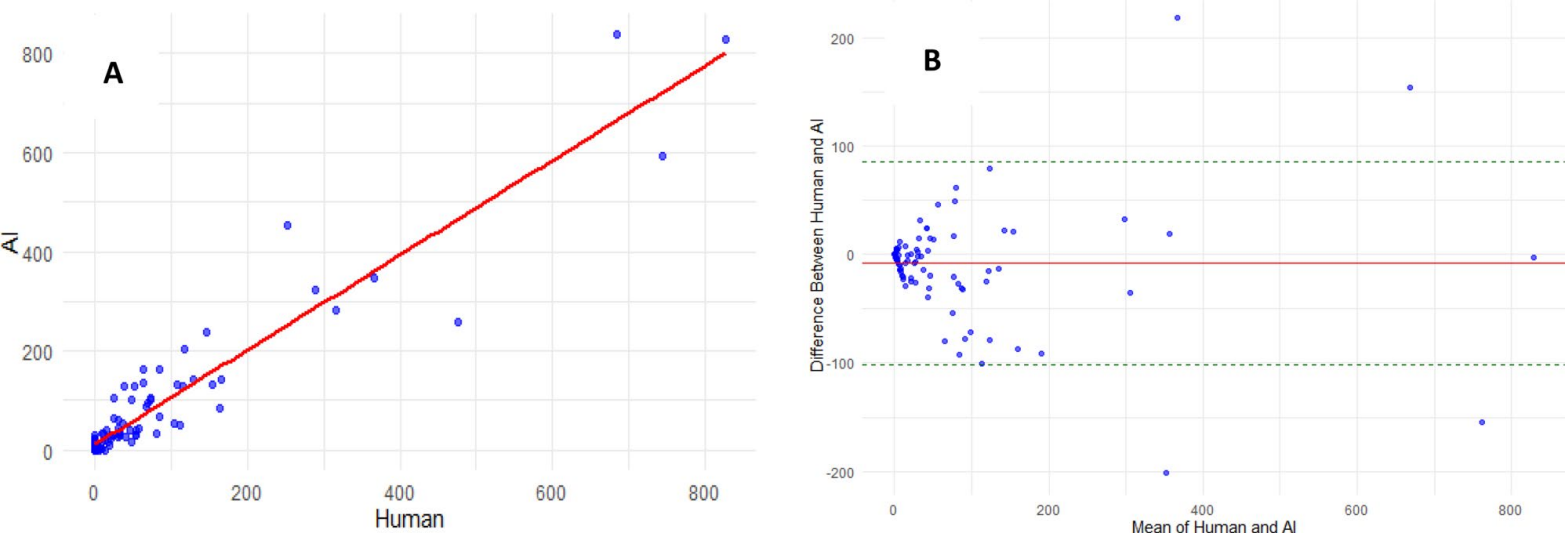
Total plaque volume. **A**: Correlation of total plaque volume between expert reader and deep learning model. ICC 0.95, Spearman’s Rho 0.87, Pearson’s r 0.95. **B**: Bland-Altman plot – Total plaque volume (bias − 8.35 mm^3^, 95% limits of agreement − 102 to 85 mm^3^)

**Fig. 3:**
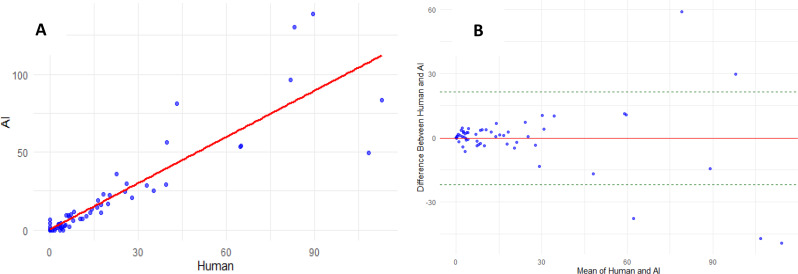
Calcified plaque volume. **A**: Correlation of calcified plaque volume between expert reader and deep learning model. ICC 0.90, Spearman’s Rho 0.93, Pearson’s r 0.90. **B**: Bland-Altman Plot – Calcified plaque volume (bias − 0.28 mm^3^, 95% limits of agreement − 22 to 21 mm^3^)

On per-patient analysis there was a strong correlation regarding total plaque volume between expert reader and deep learning segmentation (Fig. 2A); ICC 0.95 [95% CI 0.92–0.96], mean difference − 8.35 mm^3^, 95% limits of agreement − 102 to 85 mm^3^, Spearman’s rank correlation 0.87 [95% CI 0.82–0.91] and Pearson’s r 0.95 [95% CI 0.92–0.96]. Regarding calcified plaque volume there were strong correlation and good agreement between expert reader and the deep learning segmentation (Fig. 3); ICC 0.90 [95% CI 0.85–0.93], mean difference − 0.28 mm^3^, 95% limits of agreement − 22 to 21 mm^3^, Spearman’s rank correlation 0.93 [95% CI 0.90–0.95] and Pearson’s r 0.90 [95% CI 0.86–0.93].

**Fig. 4:**
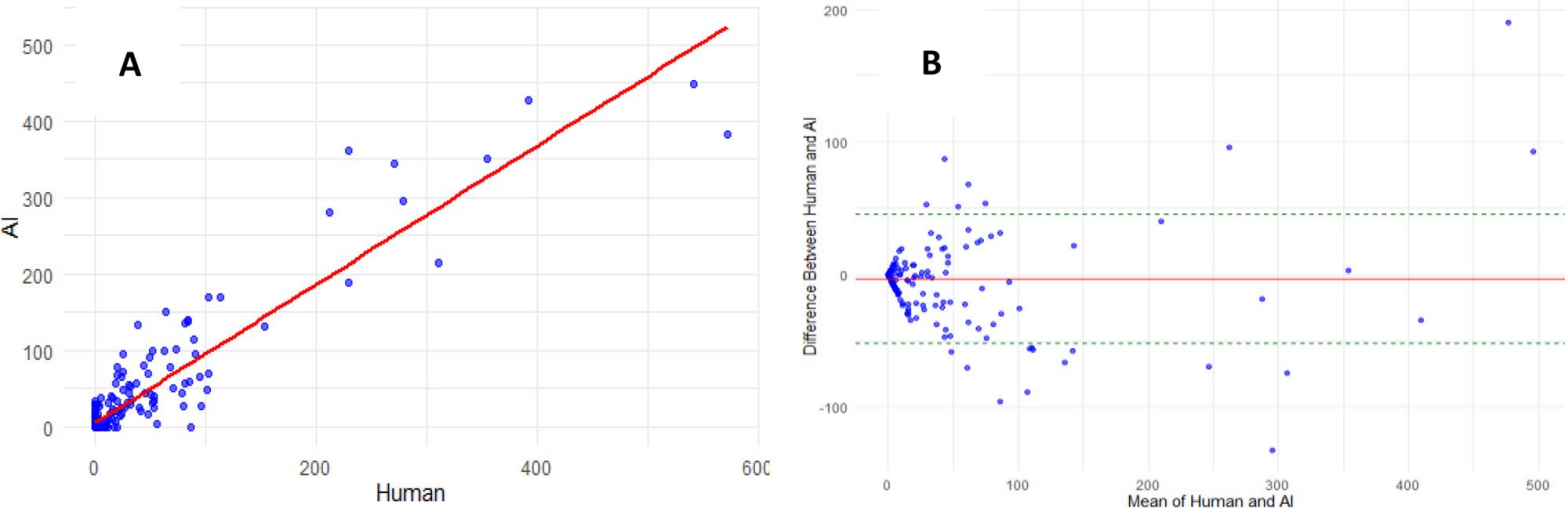
Plaque volume per vessel. **A**: Correlation of total plaque volume between expert reader and deep learning model per. vessel territory (RCA, LM/LAD, LCX). ICC 0.93, Spearman’s Rho 0.72, Pearson’s r 0.93. **B**: Bland-Altman plot - Total plaque volume per vessel

**Fig. 5:**
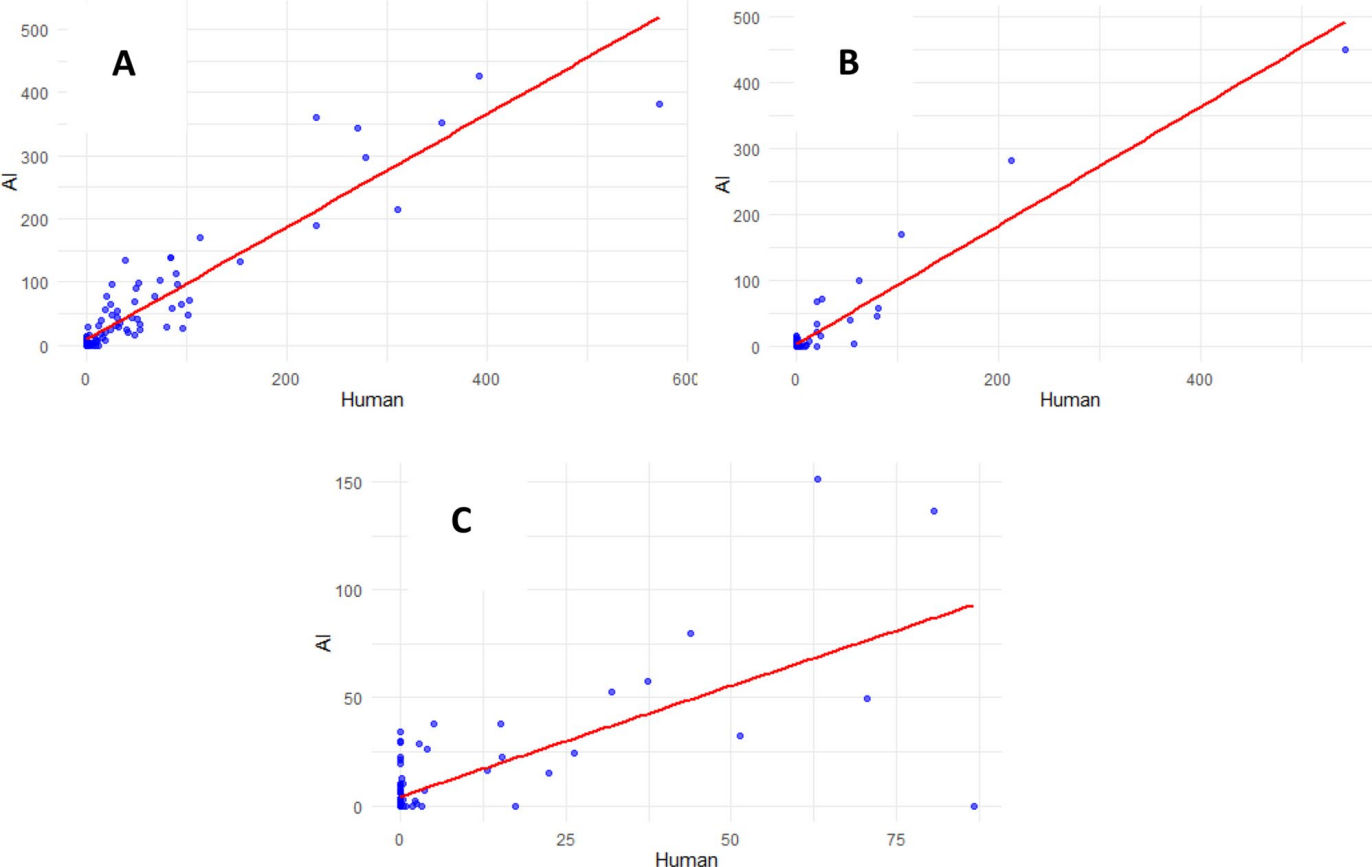
Plaque volumes in LM/LAD, RCA and LCX. **A**: Correlation of total plaque volume in LM/LAD territory between expert reader and deep. learning model. ICC 0.93, Spearman’s Rho 0.88, Pearson’s r 0.93. **B**: Correlation of total plaque volume in RCA territory between expert reader and deep learning model. ICC 0.96, Spearman’s Rho 0.54, Pearson’s r 0.96. **C**: Correlation of total plaque volume in LCX territory between expert reader and deep learning model. ICC 0.66, Spearman’s Rho 0.52, Pearson’s r 0.72

Analysis of all vessel territories aggregated showed moderate to strong correlation for total plaque volume (Fig. 4A); ICC 0.93 [95% CI 0.91–0.94], 95% limit of agreement of -52 to 46 mm^3^ (Fig. 4B), Spearman’s rank correlation 0.72 [95% CI 0.67–0.77], Pearson’s r 0.93 [95% CI 0.91–0.94]. Per-vessel analysis showed strong correlation for total plaque volume in the LM/LAD territory (Fig. 5A); ICC 0.93 [95% CI 0.89–0.95], Spearman’s rank correlation 0.88 [95% CI 0.83–0.92], Pearson’s r 0.93 [95% CI 0.89–0.95] and RCA territory (Fig. 5B); ICC 0.96 [95% CI 0.93–0.97], Spearman’s rank correlation 0.54 [95% CI 0.39–0.67], Pearson’s r 0.96 [95% CI 0.94–0.97]. Correlation in the LCX territory was moderate (Fig. 5C); ICC 0.66 [95% CI 0.53–0.76], Spearman’s rank correlation 0.52 [95% CI 0.36–0.65], Pearson’s r 0.72 [95% CI 0.61–0.80].

**Fig. 6:**
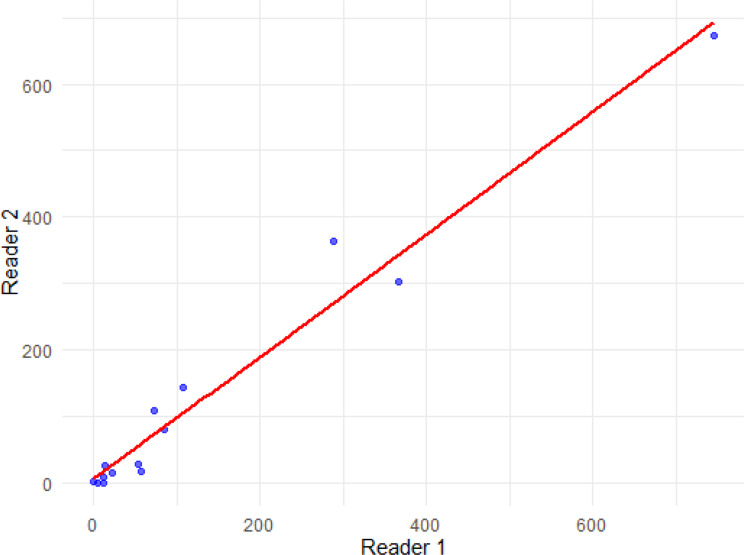
Interobserver variability. Correlation of total plaque volume between two expert readers. ICC 0.98, Spearman’s Rho 0.95, Pearson’s r 0.98

There was low interobserver variability with strong plaque volume correlation between the two expert CCTA readers (Fig. 6); ICC 0.98 [95% CI 0.95–0.99], 95% limit of agreement from − 73 to 82 mm^3^, Spearman’s rank correlation 0.95 [95% CI 0.84–0.98] and Pearson’s r 0.98 [95% CI 0.95–0.99].

## Discussion

Our results demonstrate that the developed and validated software can quantify calcified and total coronary artery plaque volume with strong correlation to and good agreement with manual expert segmentation and show performance across primary evaluation metrics comparable to reports of recent deep learning approaches for coronary artery plaque quantification [5, 13]. Lin et al. [5] report excellent agreement for total plaque volume (ICC 0.96), which is comparable to the agreement observed in the present study (ICC 0.95). In contrast to the broader scope of their study, our work is limited to external validation of total and calcified plaque volume quantification and does not include stenosis assessment or prognostic evaluation.

Correlations were strong and agreements were good also when analysing plaque volume per patient in the LM/LAD territory and RCA territory, while correlation and agreement between human reader and deep learning network were moderate in the LCX territory. In particular, there were cases with positive findings in the LCX territory by QuantiPlaque, in which the manual reader had not discovered any plaque. There may be several possible explanations for this discrepancy. Plaque in the training data were less frequent in the LCX compared to the other vessel territories, potentially contributing to a reduced model accuracy. Another plausible cause is the anatomic course of the LCX, which runs in the atrioventricular grove and often near the myocardium and fibrous atrioventricular plane with similar x-ray attenuation as the arterial wall, resulting in more challenging delineation of vessel wall outer border for both human readers and deep learning networks.

It is important to keep in mind that coronary artery plaques sometimes have diffuse borders, making precise delineation challenging for both deep learning models and human experts. Combining deep learning segmentation with human corrections may yield the most accurate ground truth. When implementing AI tools for coronary artery plaque segmentation in clinical practice, we therefore believe that a human-in-the-loop approach is warranted to leverage both time efficiency and accuracy of the AI model.

Given the encouraging results of this study, our research group plans to quantify coronary artery plaque volumes in the full Swedish CArdioPulmonary bioImage Study (SCAPIS).

### Limitations

This study has several limitations. First, all CCTA scans in the external validation cohort were obtained using the same CT scanner model, which may limit the generalizability of our findings to other scanner types, manufacturers, and scanning protocols. Future studies should validate the deep learning model on multi-vendor, multi-centre datasets to ensure robustness across different imaging conditions. However, the deep learning models have been trained on a variety of CT scanners and no scans used for this external validation have been represented in the training dataset.

Second, reference annotations were performed by a single expert reader, which may limit the accuracy of the ground truth. While interobserver variability was assessed in a subset of cases, incorporating multiple expert readers for annotation could enhance the reliability of manual segmentations and provide a more robust benchmark for model performance. In addition, the manual ground truth was not validated against an independent reference modality such as intravascular ultrasound (IVUS).

## Conclusion

Deep learning models for automated or semi-automated plaque volume quantification offer a promising avenue to enhance the prognostic value of CCTA. Total and calcified plaque volumes by the deep learning-based software that was developed and validated in this study showed strong correlation and a good agreement with human expert annotations on per-patient and per-vessel analysis.

## Data Availability

The datasets supporting the conclusions of this article are available from the corresponding author upon reasonable request.

## Abbreviations

CCTA: Coronary Computed Tomography Angiography
ICC: Intraclass Correlation Coefficient
SIS: Segment Involvement Score
CACS: Coronary Artery Calcium Score
RCA: Right Coronary Artery
LM: Left Main coronary artery
LAD: Left Anterior Descending Artery
LCX: Left Circumflex Artery
SCAPIS: Swedish CArdioPulmonary bioImage Study
